# Cascading ecological effects of eliminating fishery discards

**DOI:** 10.1038/ncomms4893

**Published:** 2014-05-13

**Authors:** Michael R. Heath, Robin M. Cook, Angus I. Cameron, David J. Morris, Douglas C. Speirs

**Affiliations:** 1 Department of Mathematics and Statistics, University of Strathclyde, Livingstone Tower, 26 Richmond Street, Glasgow G1 1XH, UK

## Abstract

Discarding by fisheries is perceived as contrary to responsible harvesting. Legislation seeking to end the practice is being introduced in many jurisdictions. However, discarded fish are food for a range of scavenging species; so, ending discarding may have ecological consequences. Here we investigate the sensitivity of ecological effects to discarding policies using an ecosystem model of the North Sea—a region where 30–40% of trawled fish catch is currently discarded. We show that landing the entire catch while fishing as usual has conservation penalties for seabirds, marine mammals and seabed fauna, and no benefit to fish stocks. However, combining landing obligations with changes in fishing practices to limit the capture of unwanted fish results in trophic cascades that can benefit birds, mammals and most fish stocks. Our results highlight the importance of considering the broader ecosystem consequences of fishery management policy, since species interactions may dissipate or negate intended benefits.

## Abstract (web summary)

Discards from fishing vessels are food for scavenging species, so ending the practice may have ecological consequences. Here, Heath *et al*. show that improving selectivity so that unwanted fish are not caught, achieves conservation benefits, while simply requiring that vessels land their entire catch, does not.

## Graphical abstract

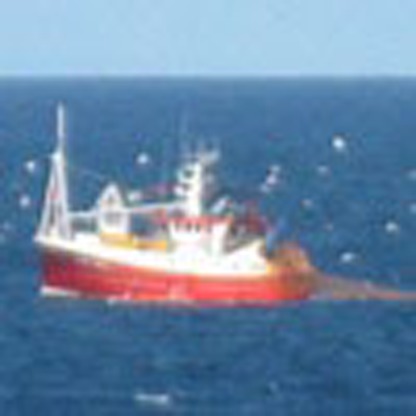

Food subsidies to wildlife as a result of human activity are recognized as having an important effect on terrestrial and aquatic ecosystems^1^. Intentional discarding at sea by commercial fisheries of unwanted fish that have little or no market value on account of size or species, or which are in excess of landing quotas, is recognized as one of the major global subsidies. The practice of discarding has been largely outside any form of regulation in the majority of fishery jurisdictions^2^,^3^. However, it is widely regarded as a waste of living resources^4^,^5^, and public opinion campaigns have pressed for changes in policy to limit discarding^6^,^7^,^8^,^9^. Norway adopted a landing obligation for cod and haddock in 1987, extending to the majority of species in 2009 (ref. 10), and in February 2013, the EU Fisheries Council voted to progressively introduce similar measures^11^,^12^. The question is how should such a policy be implemented to optimize social, economic and ecological benefits?

To address the ecological consequences of a change in discarding policy, we need to consider both direct effects on scavenging species, and the cascading of indirect effects through the entire food web; the network of species interconnected by predator–prey relationships. Enforced changes in species or resource abundances propagate through the web as a ‘trophic cascade’^13^,^14^. Fisheries cause ‘top-down’ cascades in aquatic ecosystems^15^,^16^,^17^—as a simplistic illustration, depletion of fish abundance releases herbivorous zooplankton from predation and so their abundances increase, and this in turn causes increased grazing on microalgae and so their abundance decreases. Typically, the effect is diminished with each successive trophic level. This pattern of attenuated and alternating changes in abundance between adjacent pairs of trophic levels is characteristic of a top-down cascade. Conversely, nutrient inputs at the base of the food web have a ‘bottom-up’ cascading effect leading to directly correlated changes at all trophic levels^14^. Dead or fatally damaged fish discarded by fishing vessels are a food resource for a range of scavenging seabirds, mammals, fish^1^,^18^,^19^,^20^,^21^ and seabed-living (benthic) invertebrates^22^,^23^. Eventually their remains are decomposed to release dissolved inorganic nutrients and recycled to the food web through primary production. So, we might expect curtailment of discarding to have some form of ‘bottom-up’ ecological effects on the food web by reducing food supply at various trophic levels. However, what will be the magnitude of these effects and how might they compare with the effects of changing the selectivity of fisheries so that unwanted fish are no longer captured?^3^,^12^

The North Sea is a relevant region in which to study the ecological effects of discard regulations. It is a prime example of a heavily exploited continental shelf ecosystem with well-documented landings by both pelagic and demersal fisheries^20^,^24^,^25^. Here, ‘pelagic’ refers to the group of species such as herring, sprat and sandeel that feed mainly on plankton; ‘demersal’ to species such as cod, haddock and plaice that feed mainly on other fish and/or benthos. Data collected by observers aboard fishing vessels^26^,^27^,^28^ show that the discard rate—the proportion of fish catch that is discarded—has remained relatively constant at around 30–40% by weight for the main demersal fish species (cod, haddock, whiting and plaice) since the 1970s, although the quantities discarded have declined due to diminishing catches^28^. Discard rates of pelagic fish are lower at around 10% (ref. 29).

It is difficult to conceive of a large-scale field experiment with sufficient controls to study the cascading indirect effects of alternative implementations of landing obligation policies in a large natural ecosystem such as the North Sea. As an alternative, we investigated the sensitivity to alternative implementations using a previously validated food web simulation model (StrathE2E). The model^30^ represents the dynamics of living organisms, detritus and dissolved nutrients in the North Sea as nitrogen mass in groups of functionally similar taxa and materials^31^ rather than as individual species^32^, but spans the entire ecosystem from biogeochemistry to seabirds and marine mammals (see Methods). Maximum likelihood parameters of the model have been determined by statistical fitting to an array of observational data from the North Sea^30^, and the fitted model has been demonstrated to realistically simulate both bottom-up and top-down trophic cascades^14^.

Here we compare the stationary state of the North Sea ecosystem simulated by the StrathE2E model under ‘status-quo’ discarding rates, to simulated states under two alternative illustrative implementations of a landing obligation^12^. Our first implementation scenario is a landing obligation alone without any change in fishing rates or practices so that unwanted fish are still caught, but landing quotas are inflated to accommodate the cessation of discarding. We refer to this as the ‘discards-landed’ scenario. The second maintains the status-quo landing quotas and requires that the landing obligation is achieved by more selective fishing practices so that unwanted fish are never caught, and we refer to this as the ‘improved selectivity’ scenario. ‘Discards-landed’ results in a bottom-up cascade effect with conservation penalties for scavenging seabirds, marine mammals and seabed fauna, and no benefit to fish stocks. In contrast, ‘improved selectivity’ leads to a top-down cascade, but the details depend on whether the system is being heavily or lightly exploited. In a heavily exploited state, ‘improved selectivity’ has strong benefits for birds, mammals and most fish stocks, but in a lightly exploited system, there are penalties for these apex predators. Hence, we argue that alternative implementations of landing obligation policies can produce ecological effects, which are sufficiently different that they need to be considered alongside the practical, social and economic issues.

## Results

### Discard rates of demersal fish in the North Sea

**Figure 1:**
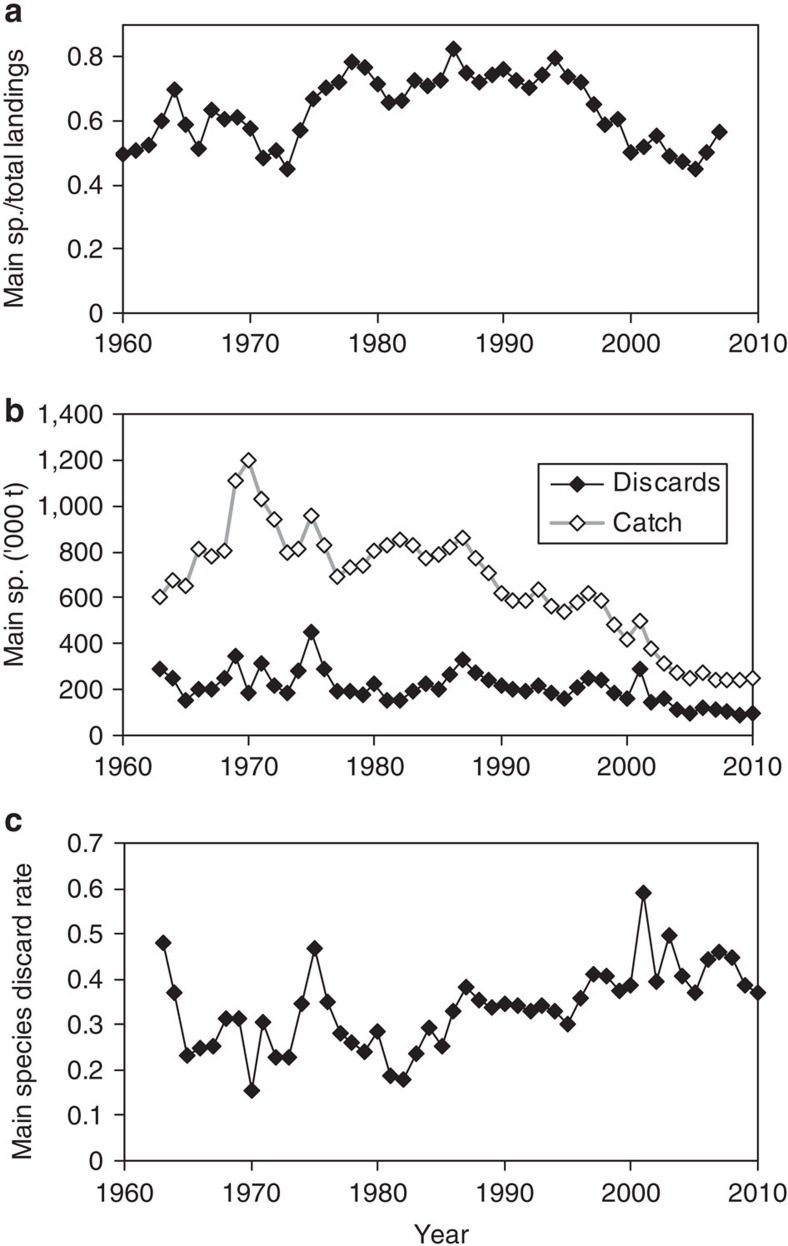
North Sea catch and discards. (**a**) Landings of the four of the main commercially exploited demersal fish species in the North Sea (cod, haddock, whiting and plaice), between 1963 and 2010, as a proportion of the total demersal fish landings of all species. (**b**) Weights of cod, haddock, whiting and plaice caught and discarded (× 10^3^ tonnes). (**c**) Proportion by weight of cod, haddock, whiting and plaice catch discarded (discard rate).

Detailed information on the quantities of all species of fish discarded by fisheries in the North Sea as a whole are available only for 1991 (ref. 29). For the demersal fish assemblage, these data indicate a discard rate (proportion of catch discarded) of 37% (ref. 29). However, four species (cod, haddock, whiting and plaice) make up around 60% of the total landed weight of all demersal species (Fig. 1). Discarded quantities of these four species are monitored annually, and have declined between the 1960s and 2010, although the discard rate has increased. The average discard rate of the combined catch of cod, haddock, whiting and plaice over period 1970–1999 was 31%.

### Harvest rates in the North Sea

**Figure 2:**
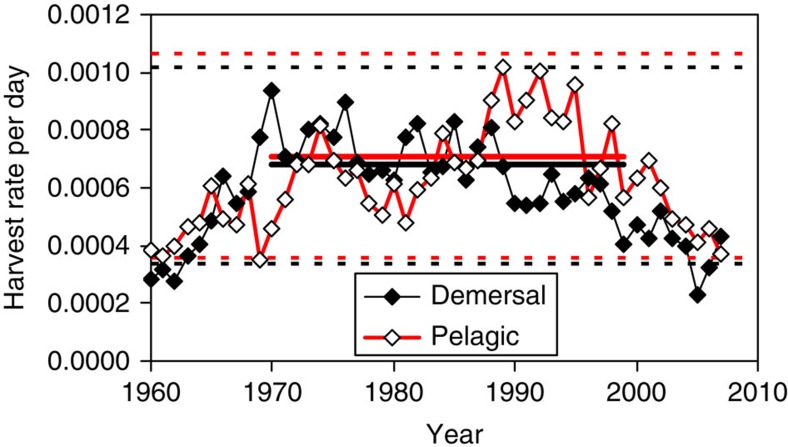
Harvest rates of fish in the North Sea. Proportions of stock biomass caught (per day) for the whole demersal fish community (black, filled symbols) and the whole pelagic fish community (red, open symbols). Baseline model rates averaged over the period 1970–1999 (horizontal solid lines) were 0.00068 for demersal fish, and 0.00071 for pelagic fish. Heavy and light exploitation scenarios (1.5 times and 0.5 times baseline) shown by horizontal dashed lines.

Biomass harvest rates (proportion of biomass removed per day) for the North Sea demersal fish community as a whole peaked at >0.0008 per day in the 1970s resulting in declining stock biomass, and were reduced to <0.0004 per day by the late 2000s as part of stock recovery plans (Fig. 2). Harvest rates for the pelagic fish community peaked somewhat later in the 1990s at >0.0009 per day and were reduced to <0.0005 per day by 2005. Average harvesting rates over the period 1970–1999 were estimated to be 0.00071 per day for pelagic fish, and 0.00068 per day for demersal fish. We refer to these as the ‘baseline’ harvest rates, since these were the rates applied to the StrathE2E model during the parameter fitting procedure.

On the basis of these data, we chose three reference conditions of harvesting under which to compare our landing obligation scenarios. The first incorporated the baseline harvesting rates corresponding to the fitted model. The other two conditions represented light and heavy exploitation conditions (0.5 times and 1.5 times baseline, respectively for pelagic and demersal fish). These encompassed the range of harvest rates that have occurred in the North Sea since 1960 (Fig. 2).

### Simulated catches

**Figure 3:**
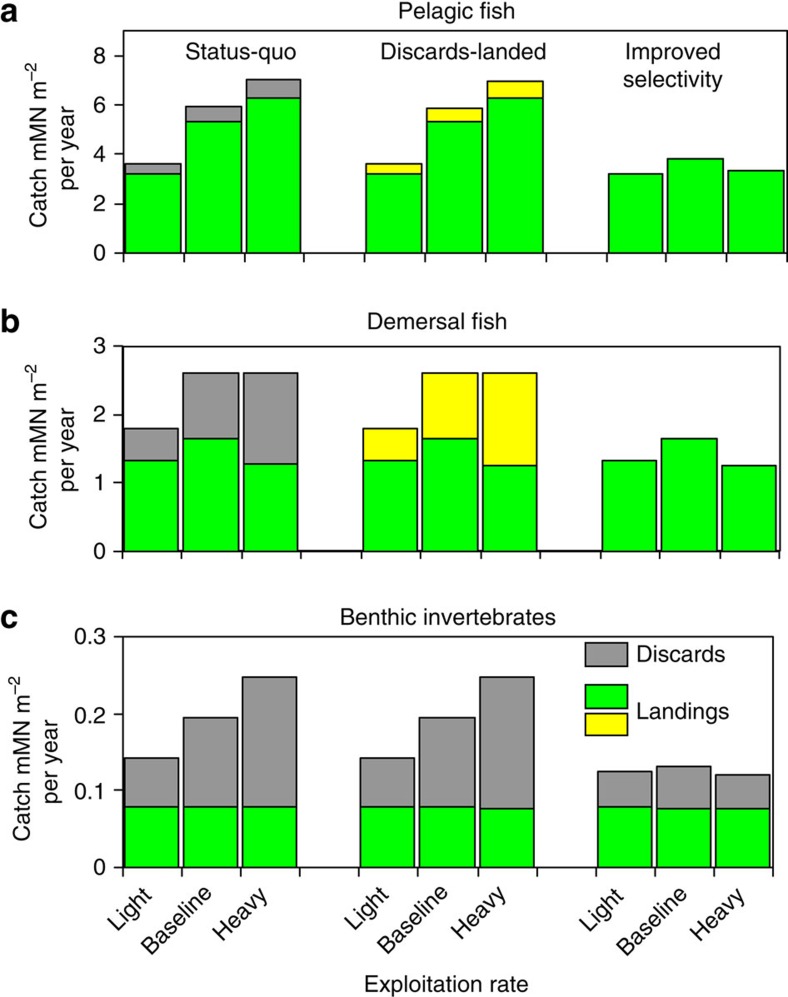
Fishery yields under landing obligation scenarios and exploitation intensities. Demersal (**a**) and pelagic (**b**) fish catches under ‘status-quo’, ‘discards-landed’ and ‘improved selectivity’ scenarios, for each level of exploitation intensity. Yellow shaded portions indicate landed components of the catch that would have been discarded under ‘status-quo’. (**c**) Benthos catch (SDB and CSB combined; see Fig. 7)—grey filled portions indicate discarded catch from both directed benthos fisheries and by-catch in demersal fisheries. Units: 1 mM N m^−2^ per year approximately equivalent to 300,000 tonnes wet weight per year of pelagic fish from the whole North Sea, 450,000 tonnes per year of demersal fish, and 1,600,000 tonnes per year of benthos^30^.

Annual catches of demersal and pelagic fish in the ‘discards-landed’ runs were indistinguishable from the corresponding ‘status-quo’ since the harvest rates were identical (Fig. 3a,b). In the ‘improved selectivity’ runs, demersal landings (rather than catches) were optimized to be equal to ‘status-quo’. To achieve this required a 58% reduction in harvest rate under baseline exploitation (80% under heavy exploitation; 34% under light exploitation).

Landings of pelagic fish in the ‘improved selectivity’ scenarios were affected by food–web interactions with demersal fish. Under baseline and heavy exploitation conditions, predation by demersal fish was increased to such an extent that the model could not achieve ‘status-quo’ landings of pelagic fish (Fig. 3a).

Discard rates of demersal fish in the ‘status-quo’ model runs were twice as high under heavy exploitation (51% of catch) compared with light exploitation (26%) (Fig. 3b). This was because the modelled demersal discard rate was parameterized to be inversely related to demersal fish biomass (see Methods and Supplementary Fig. 1). As a consequence of this, demersal fish landings under heavy exploitation were lower than under the baseline conditions despite similar catches.

With ‘status-quo’ and ‘discards-landed’ scenarios, the combined discard rate of benthic invertebrates from all fisheries, was greatest under heavy exploitation conditions (69%) compared with 45% under light exploitation (Fig. 3c). Invertebrate discard rates were substantially reduced under ‘improved selectivity’ to between 35 and 41%, due to the reductions in effective demersal finfish harvest rates (Fig. 3b,c).

### Trophic cascades

**Figure 4:**
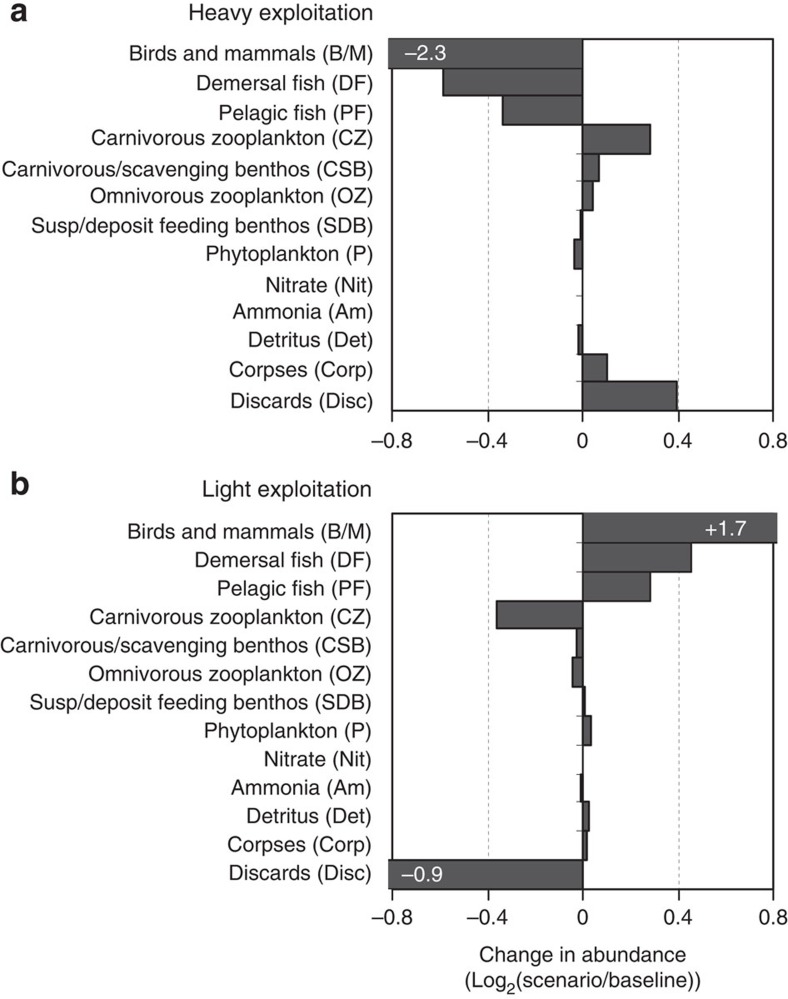
Sensitivity of food web components to the overall exploitation intensity. Horizontal bars indicate the change in stationary state abundance relative to the baseline conditions, measured as log_2_(scenario biomass/baseline biomass)^14^. Bars ranked vertically according to trophic level as in Fig. 7. Upper panel (**a**): heavy exploitation (1.5 times baseline), lower panel (**b**): light exploitation (0.5 times baseline).

Under ‘status-quo’ discarding practices, changes in fish harvesting rates alone produced cascading effects, which penetrated deep into the food web (Supplementary Figs 2 and 3). With the baseline ‘status-quo’ model as the reference, heavy exploitation caused reductions in fish biomass that cascaded down the food web producing increases in zooplankton and carnivorous benthos, small decreases in suspension/deposit feeding benthos and phytoplankton and imperceptibly small increases in nutrient concentrations (Fig. 4). Bird/mammal biomass decreased as a bottom-up response to the reduction in fish biomass. Equivalent but mirror-image results were obtained for the light exploitation results.

**Figure 5:**
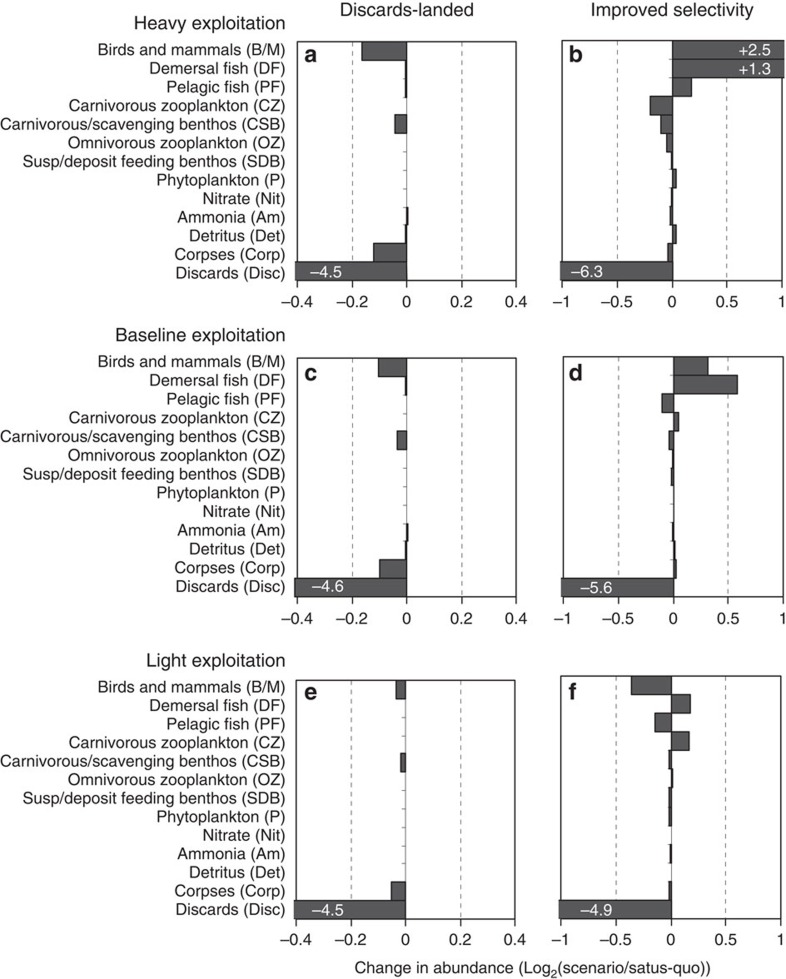
Sensitivity of food web components to landing obligation scenarios. Horizontal bars indicate the stationary state abundances of food web components under landing obligation scenarios relative to ‘status-quo’ discarding, for each of the three levels of initial exploitation intensity. Sensitivity measured as log_2_(scenario biomass/status-quo biomass^14^). Bars ranked vertically according to trophic level as in Fig. 7. Left column (**a**,**c**,**e**): sensitivity to ‘discards-landed’ scenario, right column (**b**,**d**,**f**): sensitivity to ‘improved selectivity’. Directly related changes in components of the scavenging compartment under ‘discards-landed’ indicate a bottom-up cascade. Inversely related changes between adjacent trophic levels under ‘improved selectivity’ indicate a top-down cascade.

Considering each level of exploitation separately (light, baseline and heavy) and taking the ‘status-quo’ model as the reference, the ‘discards-landed’ scenario caused bottom-up cascades—reductions in the biomasses of all the components in the scavenger compartment of the food web (carnivore/scavenge feeding benthos, birds and mammals) due to reductions in their food intake (Fig. 5). The magnitude of the effect increased with exploitation intensity in line with the ‘status-quo’ discard rates of fish and benthos.

**Figure 6:**
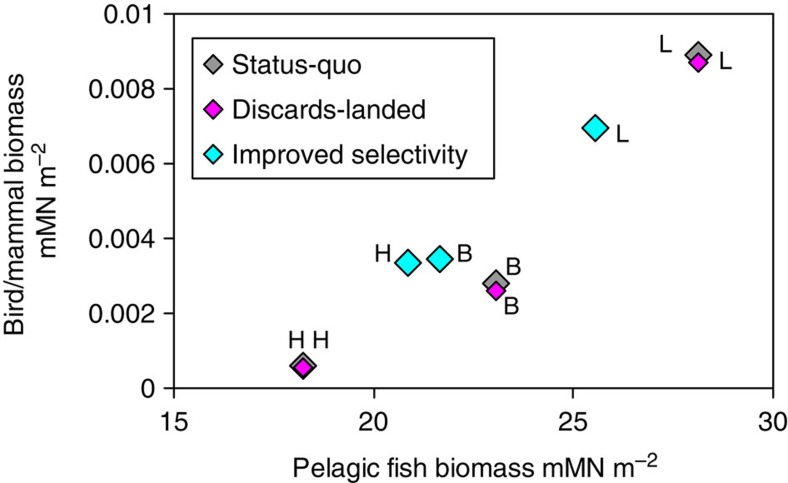
Sensitivity of bird and mammal biomass to pelagic fish. Annual averaged biomasses of birds/mammals and pelagic fish (mM N m^−2^) from each of the nine model runs (three exploitation intensities × three discarding scenarios). Grey symbols are for ‘status-quo’ discarding at the three levels of exploitation—light (L), baseline (B) and heavy (H). Magenta and turquoise symbols indicate corresponding results for the ‘discards-landed’ and ‘improved selectivity’ scenarios. Across all nine runs, *r*^2^=0.92.

Under ‘improved selectivity’, the bottom-up effects of a cessation in discarding were combined with the top-down cascade effects of the reductions in harvesting. Under light exploitation conditions, the outcome of ‘improved selectivity’ was a decrease in birds/mammals and pelagic fish, and an increase in demersal fish and zooplankton and so on. (Fig. 5). A different and larger effect (increases in birds/mammals, demersal and pelagic fish, decrease in zooplankton and benthos) occurred under heavy exploitation conditions (Fig. 5). The top-down effects on plankton and benthos were due to the combined predation patterns of demersal and pelagic fish. The bottom-up effects on birds/mammals were overwhelmingly due to variations in the abundance of pelagic fish arising from differences in effective harvest rate, rather than variations in discard rates. Pelagic fish constituted 70–80% of the food intake of birds/mammals in all model runs, and annual average biomasses of bird/mammal and pelagic fish were highly correlated (*r*^2^=0.92; Fig. 6).

## Discussion

Our purpose here was to provide insights on cascading effects of fishery landing obligation policies throughout whole ecosystems. We used modelling because controlled experiments on large-scale natural systems are impractical. Our simulations were based on a model with coarse taxonomic resolution, but which has been statistically fitted to observed data from the case study ecosystem, shows robust dynamics^30^, and generates credible representations of trophic cascades^14^. Nevertheless, this approach clearly has limitations and, for example, cannot resolve the sensitivity of particular species to changes in the availability of discards, or foraging adaptations to exploit alternative food sources^1^,^21^,^33^. Such details will require other types of models with discrete representations of species and their biological properties. Unfortunately, incorporation of such detail into whole ecosystem models does not necessarily confer greater certainty in the results. Species-specific food web models that involve more than a handful of explicitly represented species typically become excessively parameter rich^34^, exhibit fragile dynamics and are inherently difficult to statistically fit to the observed data^35^. Hence, we traded off taxonomic resolution in the model for robustness and parsimony in the number of parameters^31^.

The outcomes of our two illustrative implementation strategies are identical in terms of satisfying the social demand for discard reduction, but the ecological consequences are starkly contrasting and also depend on the initial exploitation state of the ecosystem. ‘Discards-landed’ eliminates discarding but also causes a bottom-up ecological cascade with conservation penalties, which become more severe with intensification of exploitation. On the other hand, maintaining existing landing quotas and forcing the eradication of discarding to occur through changes in fishing practices (‘improved selectivity’ scenario) leads to a top-down cascade and potential conservation benefits under heavy exploitation, but less so under light exploitation.

Our two scenarios for achieving a reduction in discards were chosen to illustrate the sensitivity of impacts on the ecosystem to grossly contrasting implementation approaches, rather than as a forecast for any specific policy initiative. Hence, they do not take into account parallel fisheries management goals such as the achievement of maximum sustainable yields or rebuilding stocks to particular biomass targets^36^. Nor do they take account of factors such as the discarding of offal resulting from evisceration at sea of large high-value demersal fish species, which also constitutes a food subsidy to scavenging species^19^. Viscera represent 5–15% of live weight depending on species^21^,^37^, and could still be legally discarded even under a total landing obligation. In addition, landing obligations in EU waters are to be progressively introduced, starting with the main quota-limited species and only extending to the majority of non-quota species after a number of years^11^,^12^. Aside from these details, our ‘discards-landed’ scenario is similar in concept to the current EU strategy for reforming the Common Fisheries Policy^11^,^12^. This involves inflation of landing quotas and creating a market for currently unwanted fish so that fishers may continue operating within the law. Alternatives involving legislative measures to improve the selectivity of fisheries are expected to be more technically challenging and economically costly both for the industry and enforcement agencies^12^,^38^.

We do not address the economic consequences of our two implementation strategies here, and they are difficult to evaluate, but they will surely be very different^12^. ‘Discards-landed’ implies that the cost of fishing will be equivalent to the ‘status-quo’, but the returns are unclear. Although there is an increase in landing quota, the market for fish that are currently discarded is uncertain, and the obligation to bring these fish ashore detracts from vessels’ capacity to transport high-value catch to markets^39^,^40^,^41^. Alternatively, we can speculate that the capital costs of implementing and enforcing ‘improved selectivity’ may be high, but the recurring operational costs thereafter may be lower than the ‘baseline’. In addition, the weight-specific value of the catch may be expected to increase under ‘improved selectivity’ as demersal fish stocks rebuild and the proportion of large, high-value fish in the catches increases^42^.

The substantial conclusion of our investigation is that fishery discards in themselves represent a relatively small subsidy to the food web, but the cascading indirect ecological effects of curbing their production can be considerable, depending on the details of how a landing obligation is implemented. Inflating landing quotas to accommodate the entire catch is a meagre solution with few conservation benefits. On the other hand, the effective reductions in the harvest rates resulting from changes in fishing practices to eliminate the capture of unwanted fish can deliver conservation benefits, especially in heavily exploited systems. These ecological effects need to be considered alongside the practical, social and economic issues in developing a sustainable policy^43^,^44^.

## Methods

### Estimation of North Sea demersal fish discard rates

Annual international landed and discarded quantities of the four of the main demersal fishery species in the North Sea (cod, haddock, whiting and plaice) between 1963 and 2010 were assembled from stock assessment reports published by the International Council for the Exploration of the Sea (ICES)^28^. Data on total annual landed weights of all demersal fish species were obtained from the ICES-FAO database^45^. From these data, we calculated (a) the annual proportion of total demersal landings accounted for by the four main species, (b) the total catch and discard quantities of the four main species combined and (c) the their discard rate (proportion of catch discarded).

### Calculation of North Sea harvest rates

Full methods for estimating biomass harvest rates of the demersal and pelagic fish guilds are given elsewhere^30^. Briefly, species biomass compositions from trawl survey data were used to extrapolate guild biomass from stock assessments of the 10 main North Sea fish species monitored by ICES^28^ (pelagic fish: Atlantic herring, Atlantic mackerel, sandeel, Norway pout; demersal fish: cod, haddock, whiting, saithe, common sole, plaice).

Annual landed weights in each guild were derived from the ICES-FAO fisheries landings database^45^. Guild landings were raised to guild catch estimates by applying an annual discard rate:


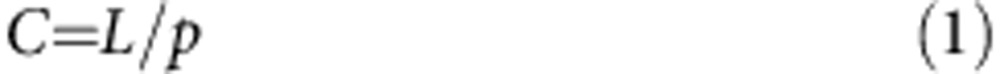


where: *C*=catch weight, *L*=landed weight, *p*=proportion of catch discaded. In the absence of comprehensive data on discarding rates, we raised the documented annual discard rates of cod, haddock, whiting and plaice^28^ to whole-guild discard rates, using a compilation of species-specific discard rates for 1991 (ref. 29). For pelagic fish, we used a single point estimate from 1991 (ref. 29). We assumed that the annual catch was taken uniformly throughout each year, and derived the daily harvest rate as the daily catch divided by the annual average biomass.

### Brief description of the StrathE2E model

**Figure 7:**
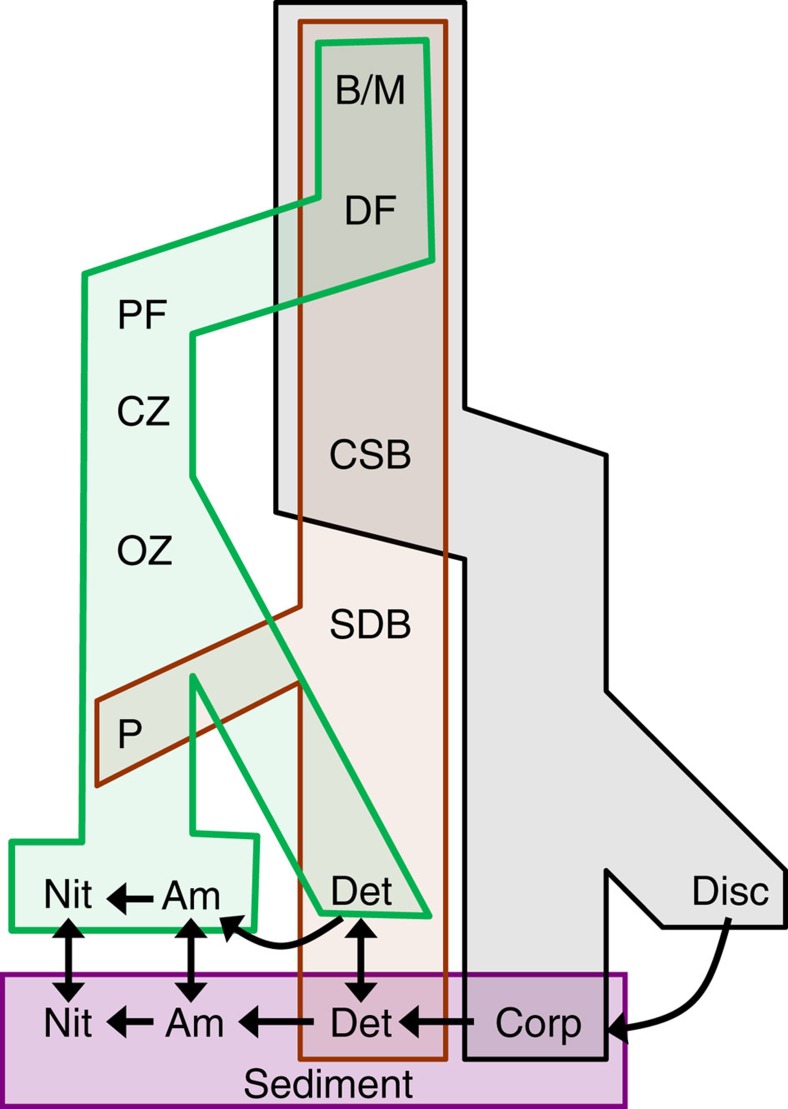
Schematic of the position of discards in compartments of the StrathE2E food web. Taxa and non-living resources in the model form three interlinked food chain compartments: grey, scavenging; orange, benthic; green, pelagic. The purple compartment represents seabed sediment geochemistry. B/M, seabirds and marine mammals; DF, demersal fish (for example, cod, haddock and plaice that feed mainly on other fish and benthos); PF, pelagic fish (for example, herring, sprat and sandeel that feed mainly on plankton); CZ, carnivorous zooplankton; OZ, omnivorous zooplankton; P, phytoplankton; CSB, carnivorous/scavenging benthos; SDB, suspension/deposit feeding benthos; Nit, nitrate, Am, ammonia; Corp, corpses; Disc, fishery discards. Omnivory occurs within each compartment, for example, PF feed on both CZ and OZ; DF and PF are subdivided into larvae and adults; Nit, Am, Det and P in the water column are subdivided into surface and deep layers. Transformations between Disc, Corp, Det, Am and Nit are due to microbial degradation, mineralization and nitrification processes. Fishery landings and denitrification represent export fluxes from the model, water column classes of P, Nit, Am and Det are subject to hydrodynamic exchanges, which generate net imports and exports depending on simulated concentration gradients. The model also includes fluxes from living components to Am, Det, Corp and Disc due to excretion, defecation, death.

Twenty-two state variables are included in the model^30^, representing the nitrogen mass (mol N m^−2^ sea surface) of classes of detritus, dissolved inorganic nutrient, plankton, benthos, fish, birds and mammals (Fig. 7). Dynamics of these variables are simulated in continuous time and output at daily intervals by integrating a set of linked ordinary differential equations describing the key physical, geochemical and biological processes that occur in the sea and seabed sediments. These include the feeding of living components, and the production, consumption and mineralization of detritus including fishery discards. Uptake of food is defined by Michaelis–Menten functions for each resource–consumer interaction defined by a preference matrix. External drivers and boundary conditions for the model are harvesting rates of fish and benthos, temperature, inflow rates of water and nutrient across the external ocean boundaries and from rivers, vertical mixing rates and atmospheric deposition of nutrients.

### Representation of fishing and discarding in the model

At each model time step, a fraction (*Fp*) of each exploited fish or benthos class in the model is transferred to the ‘discards’ state variable, and a fraction (*F(1−p)*) is exported from the model to represent the landed component of catch (*F*=proportions of biomass caught (per day) (that is, harvest rate); *p*=proportion of catch discarded (that is, discard rate)). For pelagic fish *p* is fixed at 10% (ref. 29). For demersal fish *p* is parameterized to be inversely related to biomass


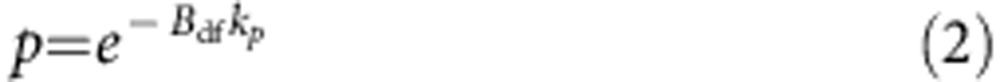


where *B*_df_=biomass of demersal fish (mM N m^−2^), *k*_*p*_=demersal discard parameter (fitted as part of the simulated annealing process). This heuristic relationship is supported by empirical evidence (Supplementary Fig. 1), and caricatures the effects of exploitation on the body size distribution of fish—lightly exploited communities should contain a higher proportion by weight of large individuals than heavily exploited communities^42^, and hence the proportion of catch, which is discarded due to small body size should be lower. For benthos classes, *P*=12% (ref. 29), while benthos by-catch in demersal fisheries is assumed to be 100% discarded. Discards have a short half-life in the model, decaying at a fixed daily rate (~\n0.4 per day) to ‘corpses’, then at temperature-dependent rates to seabed sediment detritus, and ultimately to sediment pore-water ammonia.

### Model parameter fitting

Simulated annealing^46^,^47^ was used to fit parameters of the StrathE2E model so as to minimize the discrepancy between the stationary annual cycle of the model and data on monthly and annual averaged abundances of state variables, production rates and feeding fluxes in the North Sea ecosystem, averaged over the period 1970–1999 (ref. 30). During fitting, the model was driven by 1970–1999 average annual cycles of environmental conditions and harvesting rates of demersal and pelagic fish and benthic invertebrates^30^. We refer to this fitted model and driving data, as the ‘baseline’. Details of the minimized discrepancy between outputs from the baseline model and each element of the observed data^30^ are given in Supplementary Fig. 4.

### Definition of model landing obligation scenarios

In the ‘discards-landed’ scenario, the harvest rates of pelagic and demersal fish were as in the ‘status-quo’ model but no discarding of fish was allowed so that the entire catch was landed. In the ‘improved selectivity’ scenario, no discarding of fish was permitted but in addition, the demersal harvest rate was reduced to caricature the use of more selective gears and judicious timings and locations of fishing so as to avoid the capture of unwanted fish^3^,^39^. ‘Improved selectivity’ harvest rates were optimized so that annual demersal fish catch was equal to landings in the corresponding ‘status-quo’ model, and pelagic catch was as close as possible to ‘status-quo’ landings. In both scenarios, a fixed proportion of the demersal harvesting rate was applied to benthos classes to generate a by-catch of seabed invertebrate fauna, all of which was assumed to be discarded. The harvest rates and discard proportions in the directed benthos (for example, prawn, crab, scallop) fisheries remained unchanged from the ‘status-quo’ in all cases.

### Trophic cascade patterns

We measured the proportional response of any given food web component (*i*) to a harvesting or discarding scenario, by comparing the stationary annual average biomass (B_i_*) resulting from a reference run of the model, with results from a run with altered harvesting or discarding parameters^14^:


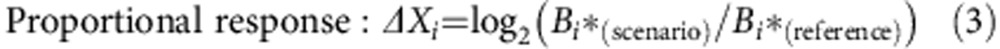


Here, *ΔX*_*i*_=0 signifies no change in component *i* as a result of the change in forcing factor value, while a value of −1 indicates a halving, and +1 a doubling. Values of *ΔX*_*i*_ were then ordered by trophic level in the model and visualized as tornado diagrams.

## Author contributions

M.R.H., R.M.C. and D.C.S. conceived the project; A.I.C. and D.J.M. carried out coding and sensitivity analysis; M.R.H. carried out model runs and wrote the paper.

## Additional information

**How to cite this article**: Heath, M. R. *et al*. Cascading ecological effects of eliminating fishery discards. *Nat. Commun.* 5:3893 doi: 10.1038/ncomms4893 (2014).

## Supplementary Material

Supplementary Information Supplementary Figures 1-4 and Supplementary References
