# Supplementary material for: Cascading ecological effects of eliminating fishery discards
Source: Nat Commun. 2014 May 13;5:3893. doi: 10.1038/ncomms4893 (PMC4024762; doi:10.1038/ncomms4893)
Supplement: Supplementary Information — Supplementary Figures 1-4 and Supplementary References [file ncomms4893-s1.pdf]

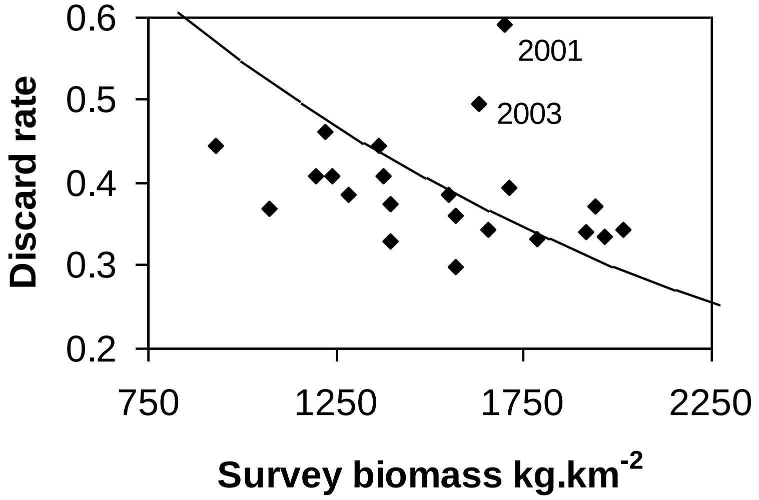

Supplementary Figure 1 | Sensitivity of demersal fish discard rates to demersal fish biomass. X-axis indicates the biomass density (kg wet weight. km^{-2}) of demersal fish estimated from catch rates during the International Bottom Trawl Surveys of the North Sea during February each year between 1989 and 2010[1]. Symbols represent the observed annual discard rates (proportion of catch discarded) for cod, haddock, whiting and plaice combined in corresponding years[2]. These four species constitute ~60% of landed weight during this period. Total community discard rate is expected to be higher since it will include a variety of lower value species[3,4], but there are no time series of total discards. Line represents the parameterised discard rate of the whole demersal fish community as used in the StrathE2E model[6] ($p = e^{-B_{df}.k_p}$; B_{df} = biomass of demersal fish ($mMN.m^{-2}$), k_p = demersal discard parameter (0.09041) m^2. mMN^{-1}). Note that the line is not intended to be a statistical fit to the observations. To convert survey biomass density to nitrogen molar density, we assumed that 1 $mMN.m^{-2}$ is equivalent to 746 kg $ww.km^{-2}$, and a survey biomass catchability rate of 20% (proportion of actual *in-situ* biomass in the path of the survey trawl which is retained)[5,6]. Excluding the two outliers in 2001 and 2003 due to extreme discard rates of haddock and whiting respectively, the observed data show a declining discard rate with increasing community biomass, as assumed in the model.

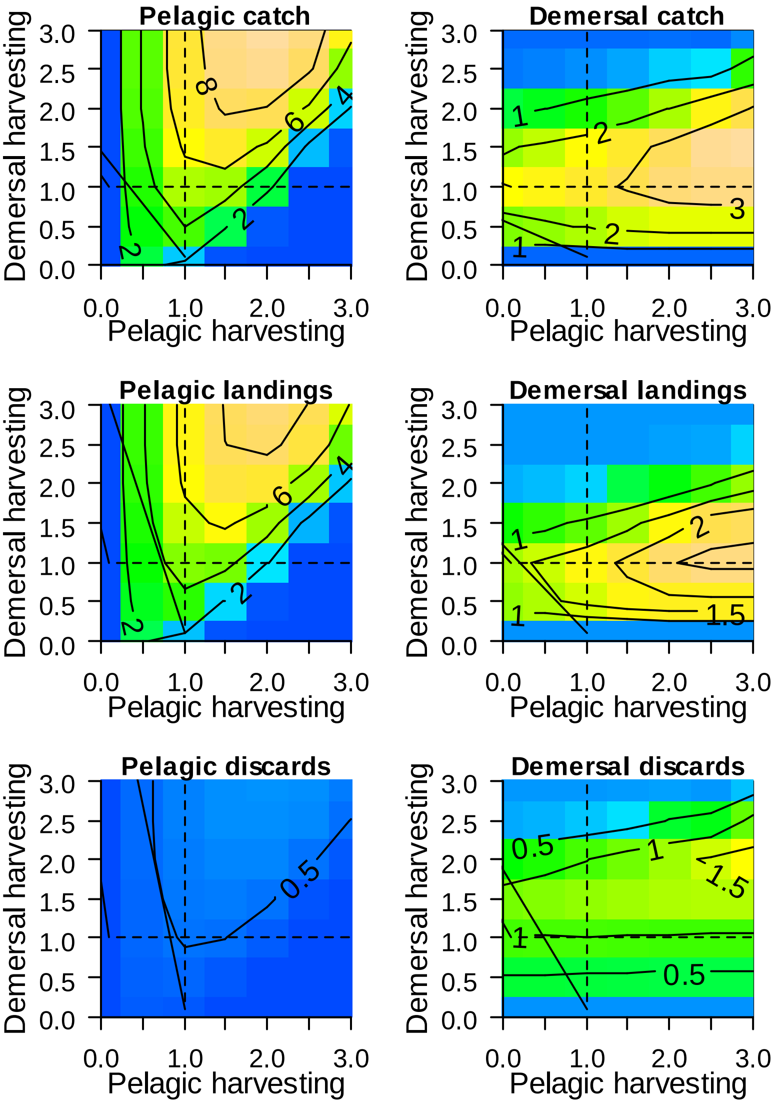

Supplementary Figure 2 | Sensitivity analysis of fishery catch in the fitted model. Each panel represents a 7 x 7 matrix of combinations of pelagic and demersal fish harvest rates applied to the fitted StrathE2E model[5]. Harvest rates are scaled relative to the 'baseline' model rates (0.00071 d^{-1} for pelagic fish; 0.00068 d^{-1} for demersal fish), so the intersection of the dashed 'cross-wires' at 1,1 represents the 'baseline' model. Left column, pelagic fish catch, landings and discards; right column, same for demersal fish. Contour line units: mM N m^{-2} y^{-1}; note that the colour scale ranges differ between pelagic and demersal. Maximum sustainable yield (MSY) is represented by the crest of the ridge running bottom-left to top-right through the pelagic catch and landings diagrams, and left to right through the demersal diagrams. Hence, the magnitude of pelagic MSY increases as demersal fish are more intensively harvested due to predation and food-web interactions Similarly, the magnitude demersal MSY increases as pelagic fish are more intensively harvested.

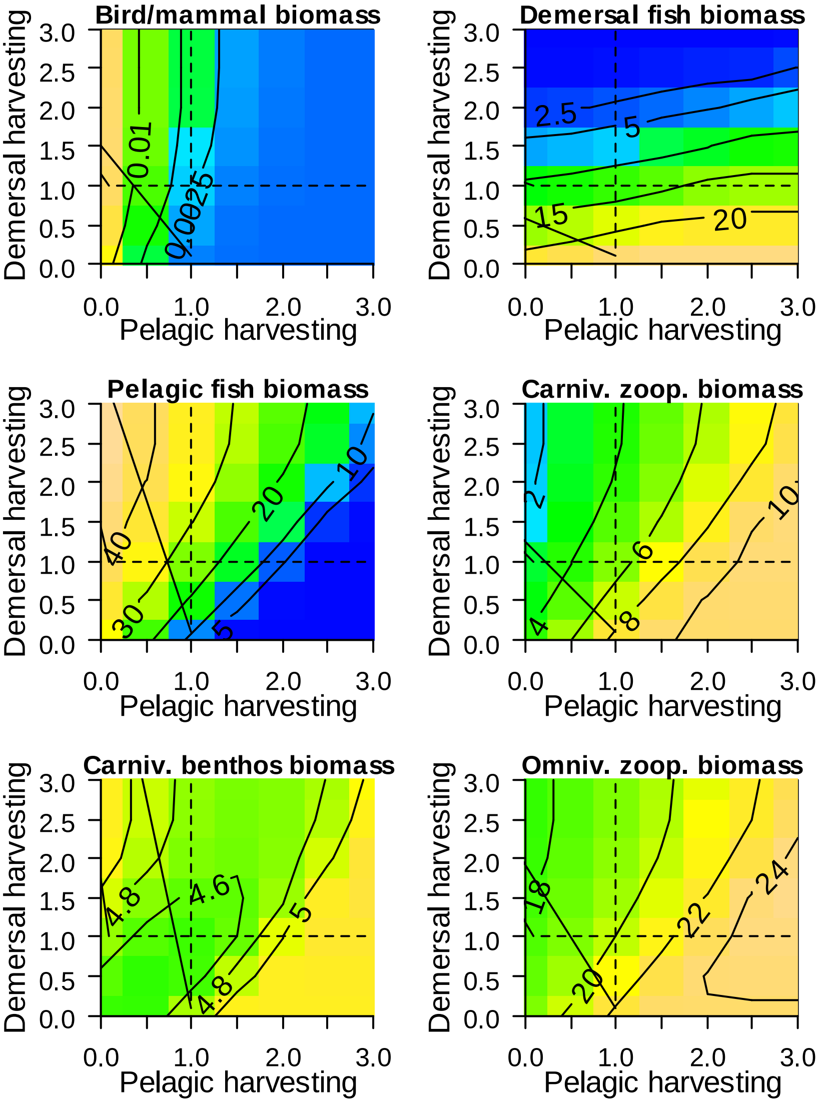

Supplementary Figure 3 | Sensitivity analysis of ecosystem components in the fitted model. Each panel represents a 7 x 7 matrix of combinations of pelagic and demersal fish harvest rates applied to the fitted StrathE2E model[5]. Harvest rates are scaled relative to the ‘baseline’ model rates (0.00071 d^{-1} for pelagic fish; 0.00068 d^{-1} for demersal fish), so the intersection of the dashed ‘cross-wires’ at 1,1 represents the ‘baseline’ model. Contour line units: annual average biomass mM N m^{-2}; note that the colour scale ranges differ between panels.

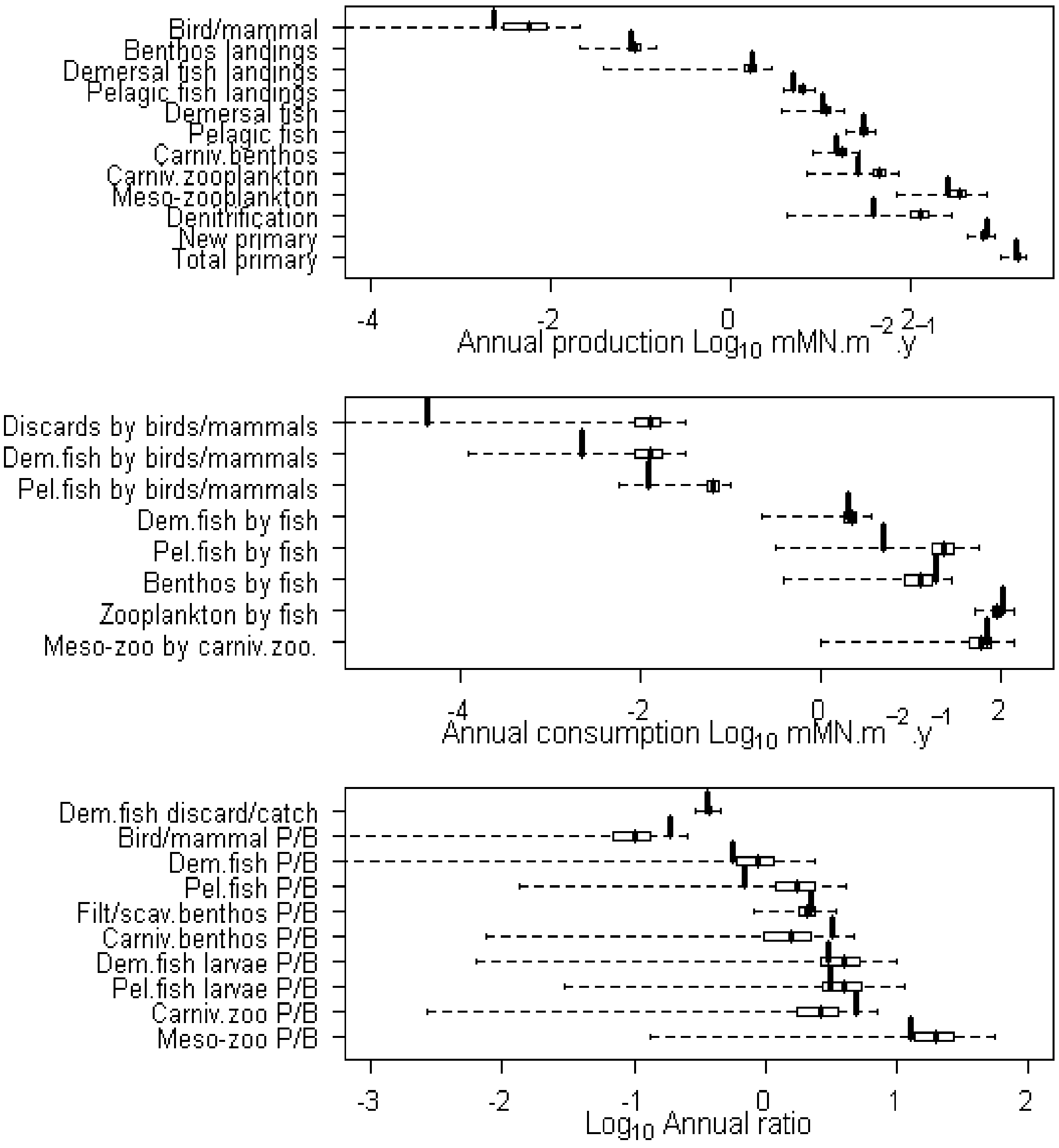

Supplementary Figure 4 | Residual discrepancies between the best-fit model and observed data. The three panels show different categories of observed data from the North Sea – annual production, annual consumption, and annual ratios. Within each panel, each row is a discrete metric averaged over 1970-1999 where possible. Box and whiskers show the range, quartiles and median over the period, or a nominal estimate of variation where no firm data exist. The vertical tick-mark above each box and whisker indicates the corresponding value from the best-fit model as a result of parameter optimisation by simulated annealing[5].

Supplementary References

1. ICES. Report of the International Bottom Trawl Survey Working Group (IBTSWG). ICES CM 2010/SSGESST:06, 261 pp. (2010). Data available from the ICES DATRAS data centre (http://www.ices.dk/marine-data/data-portals/Pages/DATRAS.aspx)
2. ICES. Report of the ICES Advisory Committee 2011. ICES Advice, 2011. Book 6 North Sea, 366 pp. (2011).
3. Mackinson, S. & Daskalov, G. An ecosystem model of the North Sea to support an ecosystem approach to fisheries management: description and parameterisation. Scientific Series Technical Report 142, Cefas Lowestoft, 195pp (2007).
4. Kelleher, K. Discards in the world's marine fisheries: an update. *FAO Fisheries Technical Paper* **470**, Food and Agriculture Organization of the United Nations (2005).
5. Heath, M. R. Ecosystem limits to food web fluxes and fisheries yields in the North Sea simulated with an end-to-end food web model. *Prog. Oceanogr.* **102,** 42-66 (2012).
6. Fraser, H. M., Greenstreet, S. P. R. & Piet, G. J. Taking account of catchability in groundfish survey trawls: implications for estimating demersal fish biomass. – ICES J. Mar. Sci. **64**, 1800– 1819 (2007).
